# Unlocking the Potential of Underutilized Technology: A New Paradigm for Resident Doctor Efficiency

**DOI:** 10.7759/cureus.63012

**Published:** 2024-06-24

**Authors:** Mohamed A Ridha, Gershon Ventour, James McParlin, Emma Cartner, Zarriar Khalid, Abdal Qadir Zafar, Ahmed Ismail, Stephen Ross-Thriepland

**Affiliations:** 1 Surgery, Huddersfield Royal Infirmary, Huddersfield, GBR; 2 Surgery, Bradford Royal Infirmary, Bradford, GBR; 3 Orthopaedics, Bradford Royal Infirmary, Bradford, GBR; 4 Orthopaedic Surgery, Leeds Teaching Hospitals NHS Trust, Leeds, GBR; 5 Trauma and Orthopaedics, Bradford Royal Infirmary, Bradford, GBR

**Keywords:** orthopaedic traumatology, decision fatigue, national health service, electronic health records, ortho surgery, healthcare tech, medico-legal implications, clinical documentation

## Abstract

Background

The precision of clinical documentation in trauma and orthopaedic surgery is pivotal, given its profound implications on patient care and medicolegal risks. This study assessed the impact of an autotext template intervention on the adherence of clinical documentation to the neurovascular assessment standards set by the National Institute for Health and Care Excellence (NICE) and the British Orthopaedic Association Standards for Trauma (BOAST).

Methods

Conducted at a single hospital, this observational study comprised two phases: a retrospective analysis of clinical documentation for 56 fracture patients (n=56) followed by the implementation of an autotext template and subsequent analysis of a new cohort of 57 patients (n=57). The intervention aimed to enhance documentation quality in line with NICE and BOAST guidelines.

Results

Initial findings revealed a prevalent use of the nonspecific term "NVI" (neurovascularly intact), with only 8.5% (n=5) of pre-intervention documents adhering to detailed motor function assessments and a mere 6.8% (n=4) recording limb colour. Post-intervention analysis showed a significant improvement, with 91.23% (n=52) of documents listing nerves (P < 0.001) and 96.49% (n=55) adhering to motor function documentation using the Medical Research Council (MRC) grading scale (P < 0.001). Despite these advancements, the study acknowledges potential limitations such as the Hawthorne effect and the ongoing challenge of staff rotations.

Conclusion

The autotext template intervention markedly enhanced the adherence to neurovascular assessment documentation standards, as evidenced by the substantial increases in detailed parameter reporting and supported by statistically significant P-values. This advancement highlights the necessity of equipping clinicians with practical tools to uphold high documentation standards amidst challenging clinical conditions. Future investigations should focus on the long-term sustainability of these improvements across varying medical staff cohorts.

## Introduction

In the domain of trauma and orthopaedic surgery, the propensity for errors carries with it considerable consequences, often manifesting as significant morbidity. Financial repercussions are substantial, with estimations suggesting that the fiscal burden of these errors can ascend to the magnitude of £1.2 billion [1]. This specialisation is distinguished by its litigious nature, ranking among the medical fields most susceptible to legal challenges [2]. Given this context, it is imperative to underscore the importance of meticulous documentation practices within this discipline. Such documentation must adhere to stringent medicolegal standards, ensuring that the care delivered is not only of the highest clinical quality but also legally defensible. This approach is pivotal in mitigating risk and enhancing patient safety within the realm of trauma and orthopaedic surgery.

Within the framework of the National Health Service (NHS) in the United Kingdom, the exigencies of managing an ever-increasing patient volume, particularly those requiring urgent care, have precipitated a marked escalation in the documentation responsibilities shouldered by resident physicians [3]. This imperative for rapid patient throughput, coupled with the consequent hastened documentation, fosters suboptimal documentation practices. Such practices not only compromise the integrity of medical records but also elevate the medicolegal vulnerability of clinicians, heightening the risk of litigation [4].

Moreover, this relentless workload serves as a catalyst for physician burnout, a phenomenon that has far-reaching implications for the quality of patient care. Burnout undermines the well-being of healthcare professionals, leading to diminished clinical performance and, ultimately, a decline in the standard of care delivered to patients [5]. This nexus between patient volume, documentation burden, risk of litigation, and clinician burnout elucidates a complex interplay of factors that necessitate strategic interventions to safeguard both practitioner welfare and patient care quality within the NHS framework.

## Materials and methods

Study design and setting

This observational study aimed to assess the adherence of clinical documentation practices to the specified standards outlined by both the National Institute for Health and Care Excellence (NICE) and the British Orthopaedic Association Standards for Trauma (BOAST) regarding the assessment of neurovascular status in patients with limb injuries [6,7]. According to NICE guideline 1.3.7, documentation should include nerve function, sensibility, and motor function using the Medical Research Council (MRC) grading system [7]. BOAST standards for supracondylar fractures further require documentation of the radial pulse, capillary refill time (CRT), and the function of the radial, median (including anterior interosseous), and ulnar nerves on presentation and immediately before any surgical treatment. Conducted in a single hospital, this study spanned two three-month phases, starting with a retrospective analysis of the clinical documentation for 56 fracture patients, followed by the implementation of an autotext template to guide documentation, with a subsequent analysis of 57 patients.

Participants

Patients who presented to the emergency department with limb fractures during the study periods were included. Exclusion criteria encompassed patients with incomplete medical records and elective orthopaedic admissions.

Intervention

To improve documentation practices, an autotext template was introduced within the hospital's electronic medical record system. This template was designed to prompt clinicians to adhere to the comprehensive neurovascular assessment criteria as mandated by both NICE and BOAST standards, ensuring the inclusion of all necessary evaluations in the clinical documentation.

Data Collection and Analysis

Data were collected retrospectively from patient medical records, focusing on the initial and pre-surgical clinical assessments documented by the clerking doctors. The analysis targeted variables specified by the NICE and BOAST standards, including assessments of nerve function, sensibility, motor function using the MRC grading system, radial pulse status, digital CRT, and the function of the radial, median, and ulnar nerves.

The R programming language was utilised for data analysis. Descriptive statistics were applied to quantify the level of adherence to the documentation standards outlined by NICE and BOAST before and after the autotext template intervention. Comparative analysis was then conducted to evaluate the intervention's effectiveness in enhancing the quality of clinical documentation.

## Results

**Figure 1:**
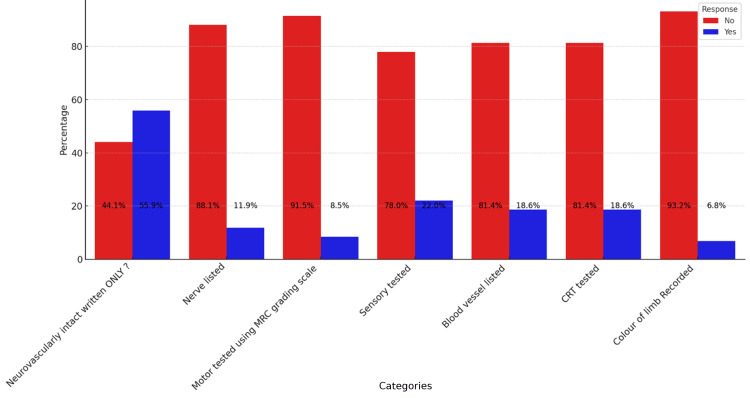
Chart demonstrating compliance to the standards prior to the intervention MRC, Medical Research Council; CRT, capillary refill time

The analysis shown in Figure 1 underscored a prevalent trend where the term ‘NVI’ or ‘neurovascularly intact’ was documented in 55.9% of cases, suggesting a reliance on this general phrase rather than adherence to detailed assessment standards. This practice contrasts sharply with the notably lower documentation rates for specific parameters such as ‘Motor tested using MRC grading scale’ (8.5%) and ‘Colour of limb recorded’ (6.8%). Other essential components such as ‘Nerve listed’, ‘Sensory tested’, ‘Blood vessel listed’, and ‘CRT tested’ were documented in only 11.9%, 22.0%, 18.6%, and 18.6% of cases, respectively. This pattern indicates a significant gap between the documentation practices and the detailed standards set by guidelines.

Our study spanned a period including August, a key transition month for new doctors. Notably, the induction training, emphasising the performance and documentation of neurovascular assessments, was uniformly administered to both new arrivals and the existing medical staff. This aimed to ensure consistent clinical documentation practices across all staff levels.

The chi-square test of independence was used to analyse adherence to documentation parameters, with the results summarised in Table 1.

**Table 1:** Results of a chi-squared test with a post-hoc Bonferroni analysis to demonstrate no statistically significant difference between the quality of documentation undertaken by previous experienced colleagues and new colleagues joining the department despite having the same induction MRC, Medical Research Council; CRT, capillary refill time

Parameter	Chi-square statistic	P-value
Nerve listed	3.25	0.1972
Motor tested using the MRC grading scale	0.21	0.9001
Sensory tested	4.33	0.1146
Blood vessel listed	6.69	0.3530
CRT tested	0.40	0.8194
Colour of limb recorded	1.23	0.5397

Despite the standardised induction process, there is an absence of statistically significant differences in documentation practices, with all P-values exceeding the Bonferroni-corrected alpha level of 0.0071.

Post-intervention

**Figure 2:**
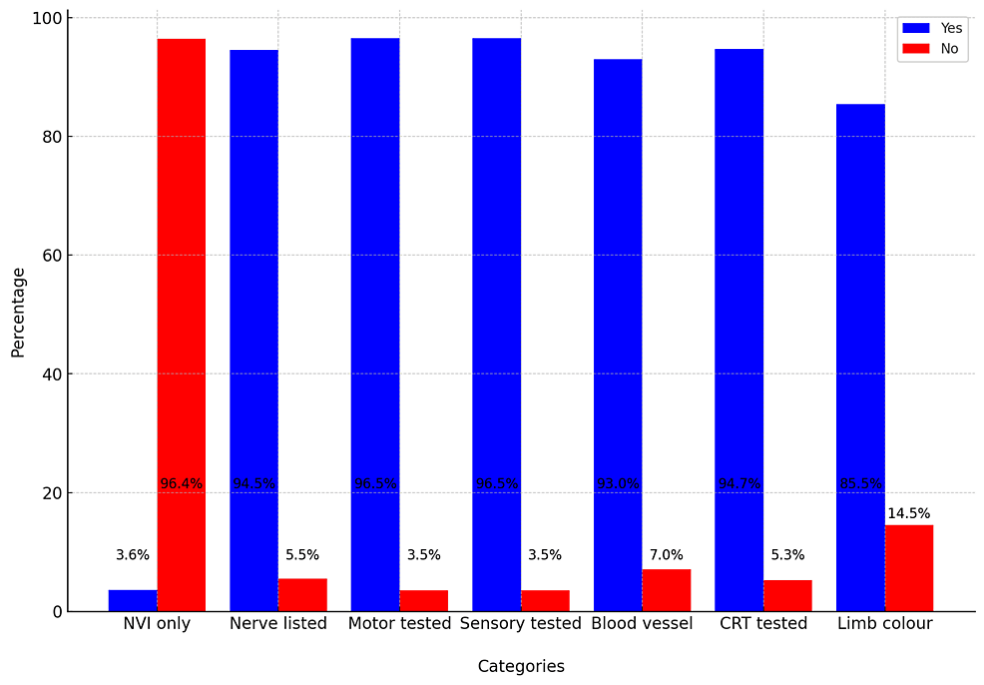
Post-intervention graph showing marked improvement on all parameters and a drastic reduction of the term ‘NVI’ NVI, neurovascularly intact; CRT, capillary refill time

In the dataset summarising post-intervention assessments in Figure 2, the percentage of affirmative (Yes) responses across different categories demonstrates a generally high adherence to assessment protocols. Specifically, 91% of entries confirmed nerves were listed, and 93% noted the listing of blood vessels. Both motor and sensory functions were tested using the MRC grading scale in 96% of cases. The CRT was tested in 95% of instances, and the colour of the limb was recorded 82% of the time.

Fisher's exact test was employed to demonstrate that the intervention yielded statistically significant results in all parameters shown in Table 2.

**Table 2:** Results of Fisher's exact test to compare the quality of documentation before and after the template intervention. All parameters demonstrated statistically significant results. MRC, Medical Research Council; CRT, capillary refill time

Category	P-Value	Pre	Post
Neurovascularly intact written ONLY?	<0.05	55.93%	3.51%
Nerve listed	<0.05	11.86%	91.23%
Motor tested using the MRC grading scale	<0.05	8.47%	96.49%
Sensory tested	<0.05	22.03%	96.49%
Blood vessel listed	<0.05	18.64%	92.98%
CRT tested	<0.05	18.64%	94.74%
Colour of limb recorded	<0.05	6.78%	82.46%

**Figure 3:**
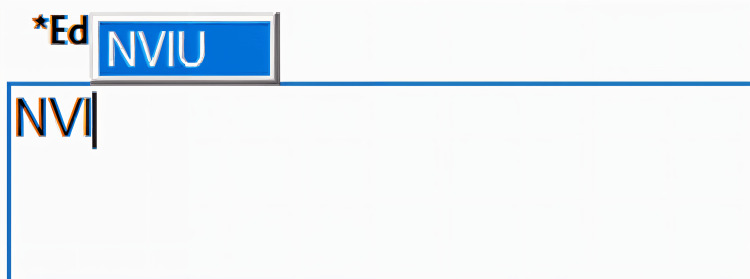
Prompt offered when someone attempts to write ‘NVI’ NVI, neurovascularly intact

**Figure 4:**
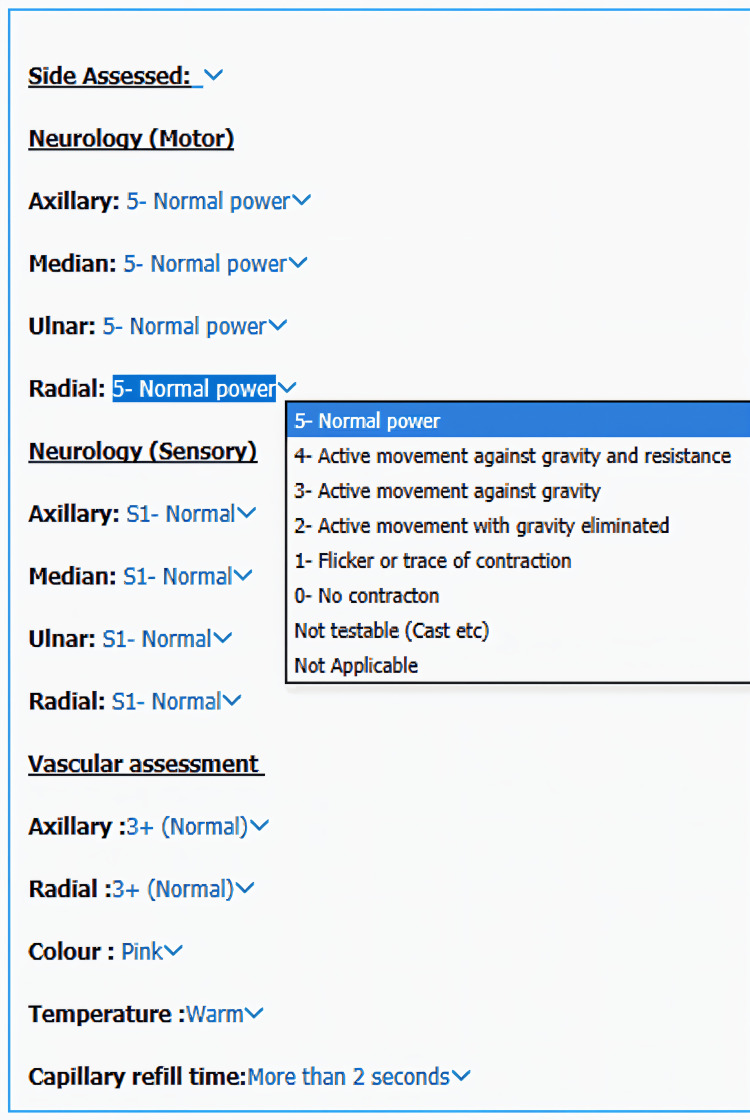
Fully populated feature showing all the parameters that need to be assessed

The intervention is as shown in Figure 3 and Figure 4. This demonstrates the prompt, which recognises that doctors write ‘NVI’, and automatically provides a fully populated template, which offers customisation to the clinician.

## Discussion

Our study identifies a critical area in orthopaedic care requiring significant improvement: documentation practices. The findings suggest that high patient throughput and limited time for detailed documentation are pervasive issues affecting all aspects of orthopaedic care.

Our results are consistent with those of McKee et al., who conducted a similar intervention [8]. They recruited 50 orthopaedic elective patients, assessing 25 patients before and 25 patients after the implementation of a new post-operative proforma at an orthopaedic elective centre [8]. They evaluated multiple parameters on day 1 post-procedure to ensure adherence to post-operative instructions. Their results indicated that prior to the intervention, critical instructions and interventions were frequently omitted. By implementing a standardised proforma, they significantly improved documentation standards across several key parameters such as neurovascular assessments [8].

This improvement can be attributed to cognitive load theory, which posits that managing a high number of simultaneous tasks can result in cognitive overload and an increased error rate [9-11]. By alleviating the cognitive burden associated with documentation through the use of proformas, efficiency is improved and errors are reduced, thereby conserving cognitive faculties [11]. Consequently, reducing the cognitive load through a standardised proforma leads to better documentation practices and a decreased likelihood of omitting important post-operative instructions [8,11].

A parallel study by Faraz et al. explored the use of educational interventions to enhance documentation standards [12]. They implemented a multifaceted approach, including posters, educational sessions, and reminders, to improve the overall quality of clinical documentation [12]. Despite these efforts, they observed that the improvement did not reach the expected standards [12]. This finding suggests that educational initiatives alone may be insufficient and highlights the necessity for additional components to achieve optimal documentation practices [12].

Among various interventions, evidence suggests that proformas outperform other initiatives [13,14]. He et al. conducted a systematic review of 28 studies, concluding that proformas improve staff satisfaction, communication, and documentation quality [13]. Bozbiyik et al. echoed these findings in their analysis of 450 operation notes, comparing different interventions, including educational initiatives similar to those used by Faraz et al. and the implementation of proformas [12,14]. Although all interventions led to improvements, they found that proformas offered the best overall outcome [14].

Despite these benefits, potential drawbacks of proformas must be considered. First, proformas can be rigid, failing to accommodate all clinical scenarios and leading to incomplete or inaccurate documentation for atypical cases. Second, there is a risk of over-reliance on the template, where clinicians might follow it without critically assessing each patient, potentially missing unique aspects of a patient's condition. Lastly, while proformas focus on structured data, they may lead to the omission of important narrative details that provide context and nuance to the patient's condition and care. They can also be cumbersome, which would then result in a worsening of clinical documentation [15].

This is demonstrated by Sibanda et al., who studied 74 patients with supracondylar fractures (48 pre-intervention and 26 post-intervention) at a hospital [15]. They found that neurovascular assessments were frequently omitted in pre-intervention patients at key points throughout their treatment journey [15]. With the implementation of a proforma, the notation ‘neurovascularly intact’ increased across pre-fracture reduction, post-fracture reduction, and post-operatively [15]. The authors attributed this to the rotational aspects of doctors' training, suggesting that the rotational aspects of the United Kingdom postgraduate training system might result in the loss of any improvement metrics observed from interventions, raising concerns about the effectiveness of a proforma and educational initiatives [12,14,15].

Sibanda et al. demonstrated that rotational doctors experience a higher cognitive burden due to adapting to new hospitals with different practices, systems, and policies [15,16]. Consequently, they are more likely to resort to System 1 thinking, a process where decisions are made quickly and automatically with minimal conscious thought [17]. The added cognitive workload of locating the proforma, placing it in the notes, and ensuring its proper use can further increase this burden, prompting doctors to switch to System 1 thinking [17]. This can result in habitual, less critical actions, potentially undermining the intended improvements of the proforma [15-17].

Faraz et al. have highlighted the effectiveness of educational initiatives in clinical practice [12]. Similarly, Sibanda et al. underscored this point [15]; however, our findings revealed no statistically significant improvement in documentation quality across our cohorts despite uniform educational induction on neurovascular assessment. This indicates that while educational initiatives hold value, they may not suffice independently, thus necessitating a more strategic approach in implementing a proforma while reducing cognitive burden. Both studies noted that the use of NVI increased in some aspects despite their intervention [12,15].

In response, we sought to leverage System 1 thinking to improve documentation practices [17]. Recognising the widespread use of the acronym NVI (neurovascularly intact) in orthopaedics [12,15,18], we developed a flexible proforma that auto-populates with all expected parameters for a neurovascularly intact patient. This design permits clinicians to tailor documentation to the individual patient, thereby alleviating cognitive load [11]. Additionally, to counteract the challenges posed by rotational training, we incorporated an explanation of each assessment to ensure precise recording of examination findings.

To our knowledge, this represents the first intervention where an in-notation prompt triggers a comprehensive assessment, thereby enhancing the detail provided by acronyms. This method maintains efficacy despite the rotational nature of resident doctor training and contrasts with traditional proformas by enabling customisation and amendments. Furthermore, by integrating directly into patient notes, it eliminates the need for separate files or pre-printed forms. This innovative approach warrants further investigation to determine its potential in maintaining the high documentation standards required in orthopaedic care.

Moreover, this intervention helps to prevent miscommunication [13,18]. Acronyms are often misinterpreted; however, this system provides clinicians with a universal definition of what constitutes a neurovascularly intact patient, without the associated documentation burden. By reducing the cognitive load on healthcare providers, this approach aims to improve the accuracy and consistency of clinical documentation.

Limitations

First, the potential influence of the Hawthorne effect, where the awareness of being observed during the study period might have inadvertently motivated clinicians to enhance their documentation practices, cannot be overlooked [19]. However, it is essential to contextualise this within the broader operational framework of our healthcare setting, where clinical documentation is routinely subject to audits. This continuous oversight mechanism serves to mitigate the transient impacts of the Hawthorne effect, suggesting that any observed improvements in documentation practices might not solely be attributed to the temporary heightened awareness but could also reflect a genuine assimilation of the intervention's principles.

Second, the dynamic nature of medical staff rotations, particularly in a teaching hospital environment, presents both a challenge and an opportunity for sustaining and evaluating the long-term efficacy of our intervention. The periodic influx of new clinicians into the orthopaedic department necessitates ongoing reinforcement of documentation standards. Consequently, the sustainability of the observed improvements in documentation practices with successive cohorts of doctors remains an area ripe for further investigation.

Future studies could focus on the longitudinal impact of our intervention, assessing whether the enhanced documentation practices are robustly maintained across successive clinician rotations and thereby contributing to a lasting culture of excellence in clinical documentation within the orthopaedic specialty.

## Conclusions

In conclusion, while the emphasis on rigorous documentation practices through educational initiatives remains a cornerstone of clinical training, the realities of high workloads and the physiological toll of sleep deprivation, particularly during night shifts, invariably lead clinicians to prioritise emergent care at the expense of comprehensive documentation. This pragmatic shift underscores the limitations of conventional educational strategies, such as posters and didactic sessions, in sustaining documentation standards under the pressures of clinical practice. It behoves us, therefore, to equip our clinicians with more than just knowledge; we must provide them with pragmatic tools designed to uphold the exacting standards of documentation expected within the orthopaedic field, even in the face of daunting clinical demands.
